# Use of Artificial Vision during the Lye Treatment of Sevillian-Style Green Olives to Determine the Optimal Time for Terminating the Cooking Process

**DOI:** 10.3390/foods12142815

**Published:** 2023-07-24

**Authors:** Miguel Calixto López Gordillo, Antonio Madueño-Luna, José Miguel Madueño Luna, Emilio Ramírez-Juidías

**Affiliations:** 1 Graphics Engineering Department, University of Seville, 41013 Seville, Spain; mlopez@us.es (M.C.L.G.); jmadueno@us.es (J.M.M.L.); erjuidias@us.es (E.R.-J.); 2 Aeroespace Engineering and Fluid Mechanical Department, University of Seville, 41013 Seville, Spain

**Keywords:** table olive, sodium hydroxide, artificial vision, data interpolation

## Abstract

This study focuses on characterizing the temporal evolution of the surface affected by industrial treatment with NaOH within the processing tanks during the lye treatment stage of Manzanilla table olives. The lye treatment process is affected by multiple variables, such as ambient temperature, the initial temperature of the olives before lye treatment, the temperature of the NaOH solution, the concentration of the solution, the variety of olives, and their size, which are determinants of the speed of the lye treatment process. Traditionally, an expert, relaying on their subjective judgement, manages the cooking process empirically, leading to variability in the termination timing of the cook. In this study, we introduce a system that, by using an artificial vision system, allows us to know in a deterministic way the percentage of lye treatment achieved at each moment along the cooking process; furthermore, with an interpolator that accumulates values during the lye treatment, it is possible to anticipate the completion of the cooking by indicating the moment when two-thirds, three-fourths, or some other value of the interior surface will be reached with an error of less than 10% relative to the optimal moment. Knowing this moment is crucial for proper processing, as it will affect subsequent stages of the manufacturing process and the quality of the final product.

## 1. Introduction

The olive tree (*Olea europaea*) is a small evergreen tree that is long-lived and can reach up to 15 m in height. It has a wide crown and a thick, twisted trunk, and its fruit is the olive [1]. The olive has been used both for oil extraction and as a food component within the Mediterranean diet [2].

The global production of table olives varies from year to year and depends on several factors, such as climate, diseases, and economic conditions. According to the International Olive Council [3], Spain produced about 2.7 million tons in the 2020/2021 campaign, which represents 20.5% of the world’s table olives, with a volume 19.3% higher than the previous year.

The industrial-scale preparation of the first table olives in Spain, “Spanish or Sevillian-style green olives”, began in the late 19th century in the province of Seville, in the towns of Dos Hermanas, Alcalá de Guadaíra, Morón, and Arahal, among others.

These Sevillian-style green table olives [4] are made from olive varieties harvested in the stage before ripening, with the desired consistency and size; the heat transfer during its cooking has been studied [4] due to the costs of the energy involved in the treatment. These costs are currently rising; thus, optimizing them is in the interest of this industry. The industrial process consists of two fundamental stages: lye treatment (to remove the bitterness due to oleuropein) [5,6] and lactic fermentation in brine. The lye treatment stage involves treating the fruits with an aqueous solution of sodium hydroxide (NaOH) at 2–4% (*w*/*v*) for about 6–12 h, depending on the variety, the ripeness of the fruits, and the temperature of the solution. Once the diffusion of the alkali has reached between two-thirds and three-fourths of the pulp, the treatment is stopped, and the NaOH solution and excess alkali are removed by one or several static water washes. Finally, the olives are immersed in a brine solution with a concentration of 10–12% (*w*/*v*) sodium chloride, where lactic fermentation takes place to prevent the growth of undesirable microorganisms and promote the growth of lactobacilli [7].

The tanks used for the lye treatment of the olives and containing NaOH and NaCl are fiberglass and polyester containers, conical at the ends and cylindrical in the central part, with a capacity of 16,000 L (approximately 10,000 kg of olives and 6000 L of alkali capacity, in the case of lye treatment tanks). The tanks are usually located inside the industry, and the lye treatment processes take place preferably at night so that the olives arrive with a lower temperature and the lye solution does not need to be cooled so much to achieve a predetermined equilibrium temperature for cooking. This represents a source of savings for the factory in refrigeration as it does not have to lower the temperature of the NaOH solution so much. The concentration of the alkali used for lye treatment is, on average, 3.0% ± 0.2% (*w*/*v*) in the tests described in this work. In current practice, as the preparation of Sevillian-style olives has extended to other varieties and, therefore, to other areas different from Seville, the range of lye concentrations used has greatly varied to maintain approximately the same number of hours for cooking.

The nutritional and sensory quality of the product depend on the cultivation conditions, mainly regulated irrigation deficits [8], pre-lye treatments (especially during transport in low-concentration alkaline solutions) [9], and the conditions of the NaOH treatment itself [10,11,12,13,14,15,16,17].

A cooking process where optimal times are not respected can lead to significant defects [7]; for example, in the Manzanilla variety, when the lye penetrates up to the middle of the pulp, the fruits remain bitter for a long time after being in brine, with the pulp near the bone having a more or less brown color. When penetration is equal to or greater than two-thirds, these issues do not occur. This phenomenon of abnormal colors, especially with olives from the Manzanilla variety from irrigated sources, is related to the greater resulting heterogeneity in the degree of maturity of these fruits, making it more difficult to determine the end point of cooking. In later stages such as packaging, these colors spread to the skin of the fruit, resulting in a loss of organoleptic quality.

Another notable example is that, if the degree of penetration of the lye is excessive, the olives break during pitting; on the contrary, if it is insufficient, the pit does not come out clean and drags a large part of the pulp.

To ensure that all olives achieve appropriate penetration simultaneously [1], the batches of this fruit designated for cooking must be as uniform as possible in size and ripeness. Once the cooking process has been completed and the lye has been removed, the olives are covered with water with the aim of removing the largest possible amount of sodium hydroxide that coats the olives and that has also penetrated the pulp. Care must be taken not to over-wash the olives to avoid loss of soluble compounds necessary for subsequent fermentation. 

The skin of the olives offers considerable resistance to the attack of the lye, with a somewhat long initial time, depending on factors such as variety, maturity, lye concentration, and temperature. During this time, the lye only acts on the skin and does not penetrate the pulp of the olive.

The washing process [7] is a complementary operation to the cooking process, and it serves to remove the lye that remains adhered to the surface of the fruits and a part of the one that penetrated inside the pulp. Another of its objectives is to partially eliminate the bitterness produced by oleuropein by hydrolysis [18].

Once the water washing is completed, the olives are placed in a brine with concentrations between 10% and 12% (*w*/*v*), where they are maintained during the fermentation and preservation stages. This fermentation process is typically conducted in buried containers, unlike the cooking process which is usually carried out in aerial ones.

A long and vigorous wash removes all traces of bitterness and lye, resulting in low pH values during subsequent fermentation, while eliminating sugars and other substances that facilitate this fermentation. A short wash produces bitter olives and high pH values that make the preservation of the olives difficult. 

If these conditions are not respected [1], problems can occur during fermentation such as (1) fissures on the exterior of the olives and interior cavities in the pulp due to the formation of CO_2_-filled blisters under the skin, a phenomenon known as “alambrado” in Spanish, (2) softening due to excessive development of microorganisms with pectinolytic activity, (3) butyric fermentation by clostridia, (4) sedimentation during packaging caused by propionic bacteria consuming lactic acid, and (5) a condition known as “zapatería” in Spanish, which occurs during conservation and is caused by clostridia.

The maximum recommended duration for the cooking and washing process is 24 h [1], although the number of washes is variable, and the current trend, considering the scarcity of water and the pollution produced by these discharges, is to give a single wash of about 12–15 h.

In the scientific literature, it is possible to find specific examples of the use of artificial vision with olives. For instance, the authors of [19] designed a system to classify olives into four categories, while also analyzing the effectiveness of various algorithms [20]. The fruit quality was evaluated and classified in [21,22] by establishing the external appearance of the skin as a determining factor for fruit quality. Some authors pointed to the use of infrared light for this purpose [23] or to estimate the ripening stage of olive lots [24]. Other authors performed high-speed inspection of olives in DRR machines using artificial vision and fast neural networks [25,26] or deep neural networks, such as Mask R-CNN [27] and CNN algorithms, or using AI algorithms and RGB imaging [28].

This article describes the application of a new system [29] that deterministically characterizes the lye treatment percentage through the use of artificial vision, as opposed to relying on the subjective judgment of an expert “maestro cocedero” in Spanish, which avoids all the problems described above. To do this, we use olive samples extracted from the tank, to which phenolphthalein is applied to emphasize the contrast between areas affected or not by NaOH. Subsequently, an image is generated and processed by artificial vision to quantify the lye treatment percentage. The obtained pairs (time and percentage) are used to feed the input of an interpolator. With the accumulated values during the lye treatment process, it is possible to anticipate its completion, indicating the moment when two-thirds, three-fourths, or another value of the surface affected by NaOH will be reached, with an error less than 10% in the estimation of the optimal moment to end the cooking process.

## 2. Materials and Method

### 2.1. Olive Variety Used

For the tests, olives from the Manzanilla de Sevilla variety [1,30,31,32,33,34,35] were used, characterized by their nearly round shape and relatively small pit, presenting a high pulp/pit ratio. It is one of the most productive and high-quality fruit varieties, which has facilitated its international spread. It presents a problem of “peeling” when treated with lye immediately after harvesting, which is why it is customary to let it rest for 2–3 days in small, well-ventilated containers [36] or, alternatively, to pretreat it with diluted lye solutions (0.5 (*w*/*v*)) for about 3–6 h.

### 2.2. Obtaining NaOH-Treated Olive Samples

**Figure 1:**
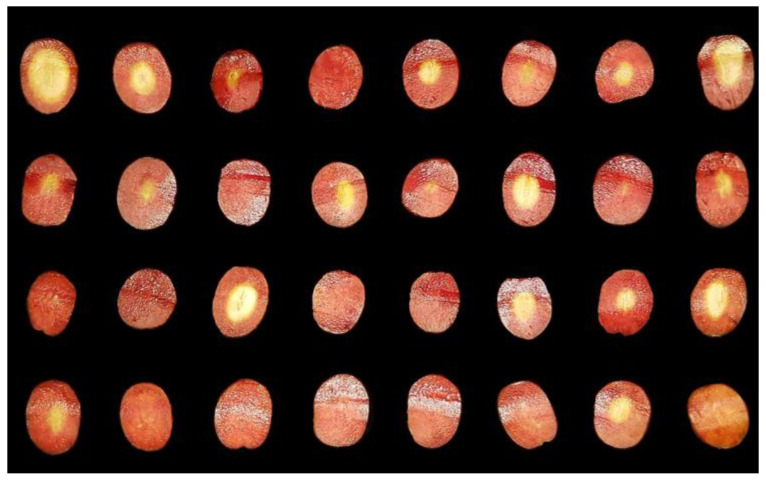
Sample of olives in 4 × 8 format colored with phenolphthalein on a dark background.

The trials were carried out in a factory in Monturque (Córdoba, Spain), along with another in Ferreira do Antalejo (Beja, Portugal), during the 2017 campaign, focusing exclusively on the Manzanilla variety from Seville. The process of cooking olives with NaOH (Brenntag Química S.A.U. Dos Hermanas, Seville, Spain), is carried out 24 h a day during the harvesting season. If performed during the night (as mentioned in Section 1), an additional advantage in energy savings is obtained. During the lye treatment process, the temperature in the tanks and the ambient temperature inside the lye treatment warehouse were monitored. Traceability was followed, indicating the concentration of NaOH, the temperature at which it was added to the tank, and the average size of the olives in the tank. Through this monitoring, the entire lye treatment process could be analyzed, from the beginning when adding the soda to the end when adding the washing water. In all cases, fine Manzanilla variety olives were used with a size of 200–210 fruits/kg, along with refrigerated NaOH (10–12 °C) at a concentration of (3–3.1% *w*/*v*). Olive samples were extracted from the tanks every hour, and the following procedure was followed: (1) cutting them axially flush with the pit, (2) applying a spray of phenolphthalein to obtain an intense coloration in the area affected by NaOH, (3) allowing them to dry for 5 min, (4) placing them in a scanner forming a matrix of four rows by eight columns (other arrangements such as 4 × 6 can also be used depending on the method employed to obtain the image of the olives; see Section 2.3), where the background is replaced by a black-colored surface, ensuring identical lighting and image size conditions between samples, Figure 1.

### 2.3. Image Capture System for Olives during the Lye Treatment Phase

**Figure 2:**
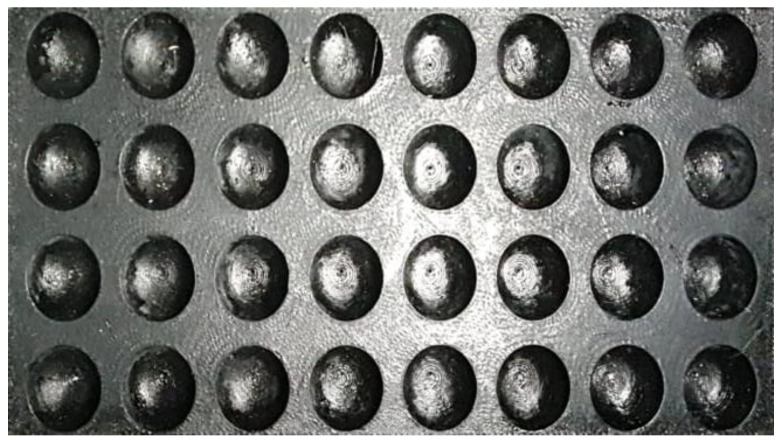
Honeycomb support designed with 3D printing to hold up to 4 × 8 olives.

The system consists of a black alveolar support designed by 3D printing and prepared to accommodate the olives; the size of the alveoli depends on the caliber of the olives (Figure 2). The black color allows contrasting the background with the olives and thus defining their contour precisely.

**Figure 3:**
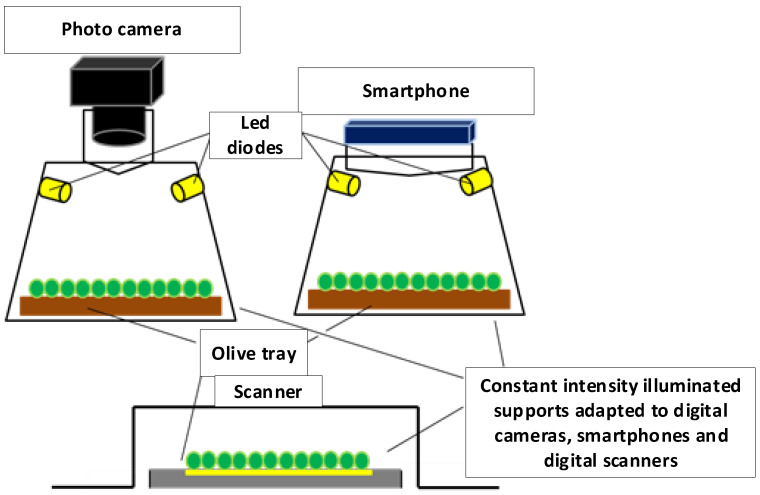
Receptacle for maintaining constant lighting (with camera, smartphone [the one we used], and scanner) and LED lighting system.

To maintain constant lighting, the support is placed inside a box whose ceiling contains an LED lighting system (Figure 3) with the ability to adjust light intensity [37], using a constant electrical intensity source [38,39].

### 2.4. Artificial Vision Analysis System

**Figure 4:**
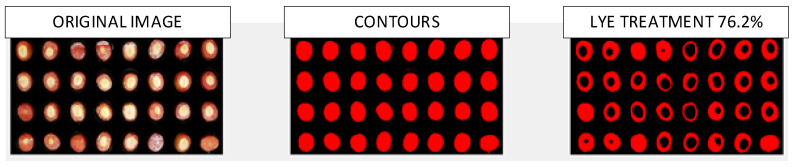
Image analysis process with artificial vision in 4 × 8 format.

For the image analysis, a procedure and software developed at the University of Seville [29] were used, through which the green and red channels of the RGB image were analyzed, as they contain information related to the penetration process of NaOH into the pulp; the blue channel was used as an element to segment the background. The images were acquired in two formats: (1) JPEG at 75 × 75 pixels per inch horizontally and vertically, respectively, and with an image size of 1500 × 1125 pixels; (2) JPEG at 150 × 150 pixels per inch horizontally and vertically, respectively, with an image size of 900 × 900 pixels, proving to be a sufficient resolution for the artificial vision determination process of the cooked surface with lye. Figure 4 shows the intermediate phases of the process for a 4 × 8 format image: segmentation of the olives from the background, identification of the part attacked by NaOH, and determination of the cooked percentage.

### 2.5. Interpolator for Predicting the Optimal Moment to End the Lye Treatment Process

**Figure 5:**
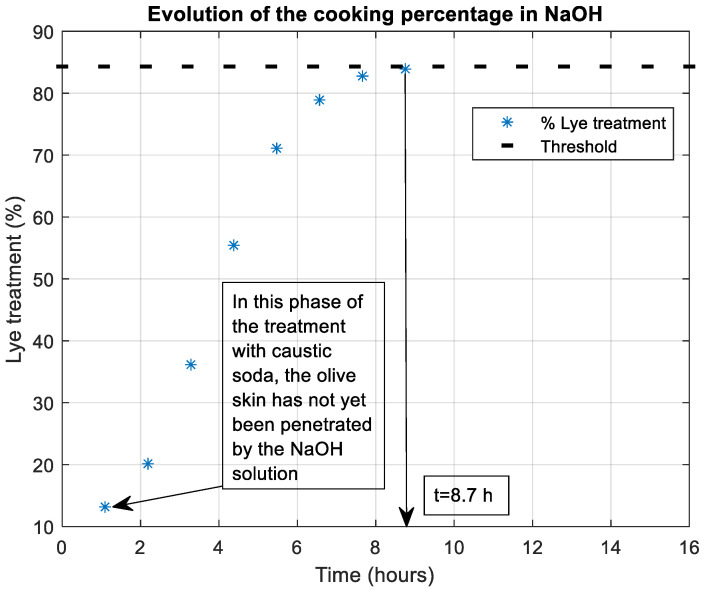
Example of how the lye treatment percentage evolves with a threshold at 84.3% when proceeding to washing with water. This sigmoid curve has an initial phase of slow growth because the olive skin has not yet been penetrated by the caustic soda.

The evolution of the lye treatment curve, as seen in Figure 5, includes the following phases: the beginning of the process with a slow speed and reduced slope, which coincides with the filling of the tank with refrigerated lye [4] (in this phase, the olive skin is permeated by the action of caustic soda); the central phase with high speed and increasing temperature; the saturation phase due to lye consumption and the end of the alkaline process with the addition of washing water. From this point on, it can be considered that the penetration speed of NaOH inside the olive decreases considerably and stops completely when the olives are poured into the fermentation brine.

To determine the optimal moment to end the lye treatment process, an interpolator [40] is used, developed using the interp1 function in Matlab R2022b [41] (Figure 5). This interpolator allows estimating, on the basis of a previously set lye treatment percentage, the necessary lye treatment time in NaOH. It is important to note that the actual end time of the lye treatment process is determined by experts according to their experience, which may vary slightly from the theoretical prediction of the preset lye treatment percentage.

The interpolation performed is based on the fact that we know two limits: first, at the beginning of the process, the lye treatment percentage is 0%; second, at the end, if the process is extended much longer than the optimal time (when the washing water is added), it is 100% (for example, after 24 h). This allows us to dynamically correct the interpolator’s prediction and get a good approximation of the completion time even from the sixth sampling.

### 2.6. Cloud Computing System for Commercial-Level Analysis during Production 

**Figure 6:**
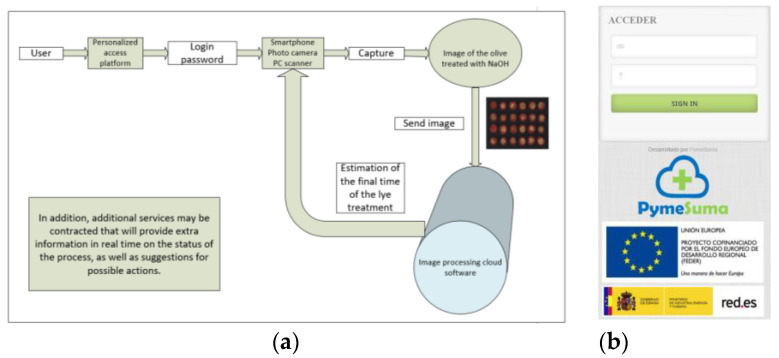
Web application for remote management of lye treatment. (**a**) Flowchart of the web application operation. (**b**) Login screen of the web application.

To manage the lye treatment process remotely and at a commercial level, a web application (lye treatment) was developed using Matlab R2022b, Glassfish 5.0, Java JDK 1.7.0, and MySQL 5.7, capable of analyzing the process online, as shown in Figure 6a,b.

## 3. Results

### 3.1. Laboratory Tests

**Figure 7:**
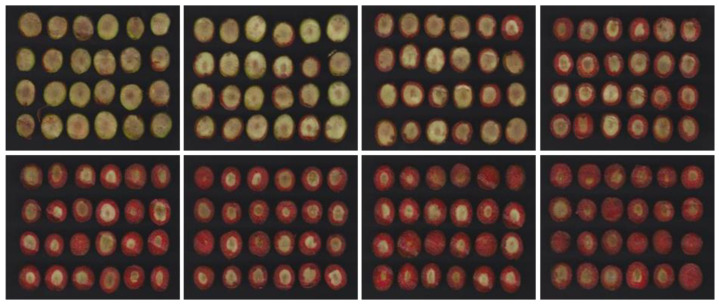
The 4 × 6 format images of olives at different moments of the lye treatment process with NaOH; the last image corresponds to the phase of washing the olives in water.

Figure 7 shows one of the cases studied for the lye treatment (4 × 6 format). The total duration of the process was 8 h, starting at 23:00 h with the addition of refrigerated NaOH and ending at 7:30 h with the final wash with water. The results shown for each sample were obtained with a 1 h interval, including the final washing of the olives with water (sample number 8 in Figure 7).

As can be seen, the procedure followed for obtaining the images ensures a constant background and lighting that allows a clear distinction between the olives and the background. The contrast provided by the phenolphthalein after the drying process allows for a perfect contrast between the area affected by NaOH and the area that has not yet been affected.

**Figure 8:**
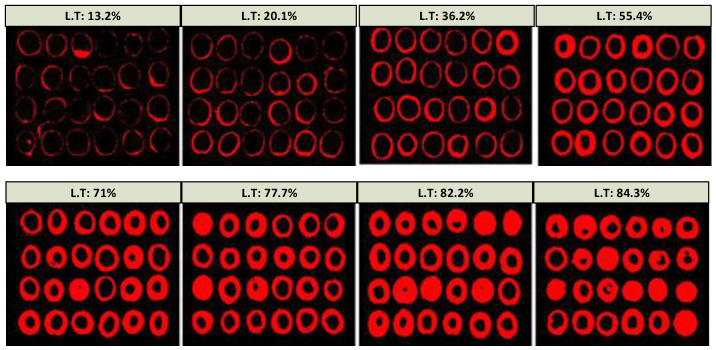
The 4 × 6 format images of olives processed with indication of the percentage of lye treatment (L.T).

In Figure 8, the images processed by the artificial vision software are shown, depicting the temporal evolution of the percentage of lye treatment.

In each trial, a total of five repetitions are performed to obtain an average value of the percentage of lye treatment at that determined time. The moment of finalization is established on the basis of the type of olive and its ripeness as main factors (for this example of an olive at the beginning of the campaign, the estimation was 85%, but it ended with the washing water at 84.3% according to the expert’s criterion). The interpolator allowed predictions of the finalization time from the first six trials with an error on the optimal time of finalization of the lye treatment process below 10%.

**Figure 9:**
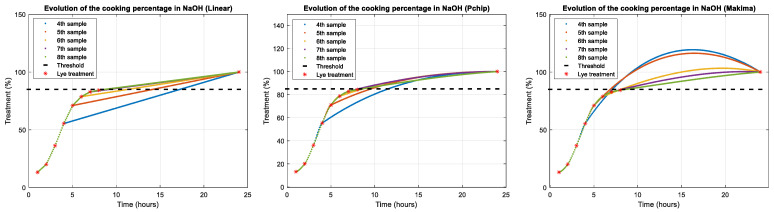
Interpolators used (Linear on the **left**, Pchip in the **middle**, and Makima on the **right**). The figure shows the evolution in the estimation of the cooking percentage according to the sample number.

The results of the progressive use of the interpolators are shown in Figure 9, depending on the interpolation method used.

For the case under study (see Table 1), it can be seen that the error in rounds 6 and 7 was less than 10% (bold and white) using interpolation methods such as Pchip and Makima, both of which can serve as estimators of the moment of completion, with the linear estimator being discarded. These data can be useful for the expert to pay attention only to the tanks that are about to finish.

As can be seen, the final error due to the discrepancy between the predicted theoretical percentage of completion and the one finally decided by the expert (bold and gray) was also within 10%.

### 3.2. Factory Trials

The tests were carried out on 23 and 24 October 2017 in Monturque (Córdoba, Spain) and on 2 November 2017 in Ferreira do Antalejo (Beja, Portugal). The data from 2017 were from research trials. The data from subsequent years were internal to the olive factories and are not publishable because they are private. The intervals between samples were those used by the expert, and the results are shown in Table 2.

As observed, only the error data regarding the final moment of the lye treatment process, fixed at the expert’s discretion, are presented. Mostly, these errors were below 10% (bold) for the interpolation methods of Pchip and Makima, always overestimating the lye treatment time for the linear case, in a ratio of 5/7 (underestimation/overestimation) for Pchip and Makima. Underestimation can be advantageous as it alerts the expert in advance.

A criterion that can be established is to carry out a new sample at around 50% of the estimated finalization time after the sixth sample.

Lastly, as shown in Table 2, the average absolute error values were below 10% (bold) for Pchip and Makima, which is a good estimate in a process that ultimately depends on the expert’s judgment for its finalization.

### 3.3. Use of the Cloud Computing Web Application

**Figure 10:**
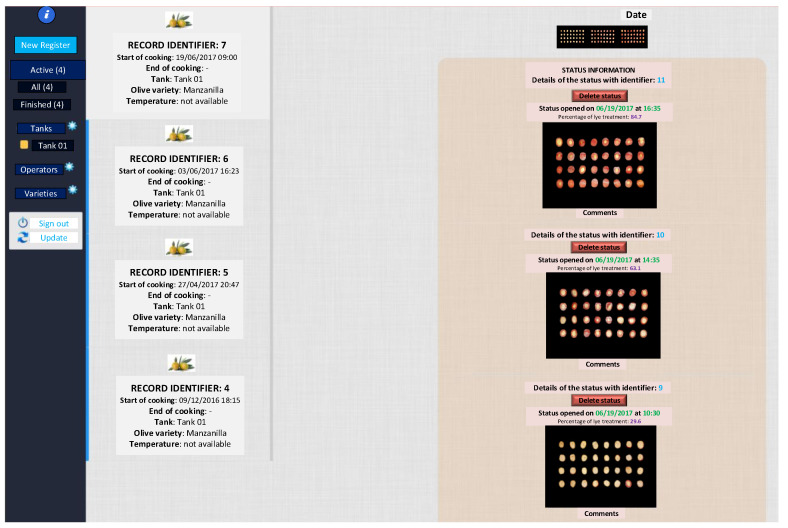
Cloud computing application (sample management).

**Figure 11:**
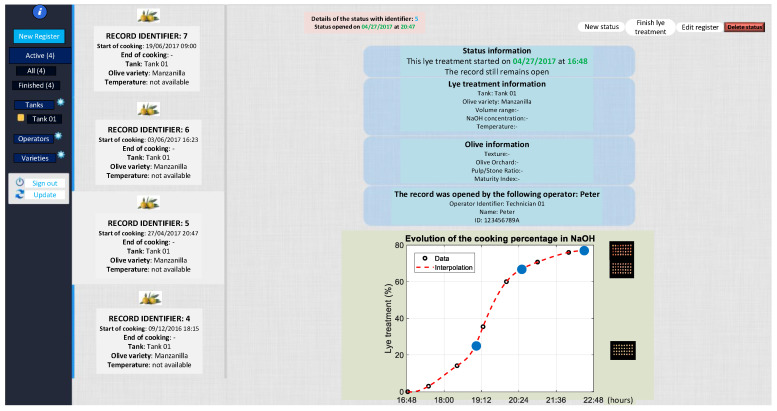
Cloud computing application (lye treatment evolution).

**Figure 12:**
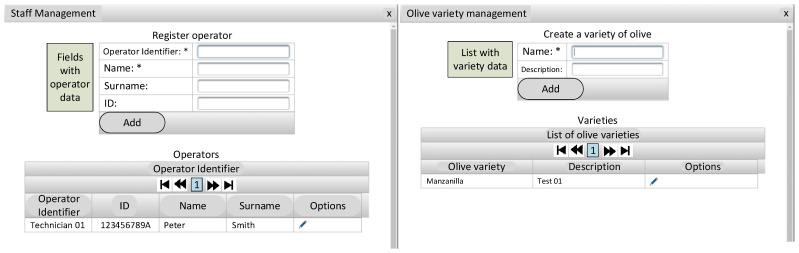
Cloud computing application (staff management and olive variety management).

Figure 10, Figure 11 and Figure 12 show the cloud computing web application. This application allows the management of samples (including parameters such as temperature or NaOH concentration used at the beginning of the lye treatment process), analysis of the sample evolution, estimation of the lye treatment finishing time, management of expert personnel, and the variety of olive used.

## 4. Conclusions

It is possible to quantitatively determine the evolution of olive lye treatment in NaOH using a previous conditioning system for the olives: (1) axial cutting flush with the pit; (2) addition of a phenolphthalein spray to obtain an intense coloration in the area affected by NaOH; (3) drying for 5 min; (4) scanning of the olives forming a matrix in different formats (4 × 8 or 4 × 6), where the background is replaced by a blue surface; (5) processing of the obtained image with artificial visions.

Using a simple interpolator, it is possible to predict the optimal moment of completion of the lye treatment, which implicitly includes information about the variety, ripeness, evolution of temperature during lye treatment, etc.

The interpolation method is more suitable than other options, such as a predictive neural network, as it requires fewer data points for adjustment, unlike the latter.

The use of a web application allows an expert to simultaneously attend to several factories and only those tanks that are near the end of the olive lye treatment process.

Although the application provides an estimate of the moment of completion of the lye treatment, the process is constrained by the experience of the expert, who knows the approximate times for each scenario (variety of the olive, ripeness, temperature, concentration, and number of uses of the soda).

If the completion time of the lye treatment is underestimated, this errs on the side of safety. Good practice would be to perform tastings at 50% of the time provided by the prediction.

With a large number of lye treatments performed, it would be possible to use a neural network for more accurate predictions or to adjust sigmoid curves.

In future work, a procedure will be tested that dispenses with the use of phenolphthalein and instead uses a thermal camera to profile the area penetrated by NaOH.

## Figures and Tables

**Table 1:** Relative errors (%) as a function of the number of samples and interpolation methods.

n_Samples	t_Lineal (h)	t_Pchip (h)	t_Makima (h)	t_Real (h)	e_Lineal (%)	e_Pchip (%)	e_Makima (%)
4	17.28	11.47	7.1	8	116	43.375	−11.25
5	14.17	9.6	6.85	8	77.125	20	−14.375
6	11.3	8.65	8.1	8	41.25	**8.125**	**1.25**
7	9.1	8.15	8.2	8	13.75	**1.875**	**2.5**
8	8.71	8.55	8.65	8	8.875	**6.875**	**8.125**

**Table 2:** Relative errors (%) obtained in the Monturque tests on 23 October 2017 and 25 October 2017, and in the Ferreira do Antalejo tests on 2 November 2017.

10/23/2017	n_Samples	t_Lineal (h)	t_Pchip (h)	t_Makima (h)	t_Real (h)	e_Lineal (%)	e_Pchip (%)	e_Makima (%)
**Coc_3**	7	7.42	6.71	6.67	6.92	**7.23**	**−3.03**	**−3.61**
**Coc_4**	7	7	5.35	5.04	4.75	47.37	12.63	**6.11**
**Coc_6**	7	6.1	5.73	5.95	6.59	**−7.44**	−13.05	**−9.71**
**Coc_8**	7	10.53	8.55	7.83	7.5	40.40	14.00	**4.40**
**10/25/2017**								
**Coc_5**	8	7.68	6.25	6.06	5.92	29.73	**5.57**	**2.36**
**Coc_8**	8	10.29	9.03	9.26	8.33	23.53	**8.40**	11.16
**Coc_9**	8	7.53	6.33	6.41	6.17	22.04	**2.59**	**3.89**
**Coc_10**	8	6.78	5.98	5.97	6	13.00	**−0.33**	**−0.50**
**11/02/2017**								
**Coc_8**	8	5.91	5.55	5.53	5.67	**4.23**	**−2.12**	**−2.47**
**Coc_9**	8	7.69	6.26	6.35	5.5	39.82	13.82	15.45
**Coc_10**	8	6.91	6.43	6.37	6.16	12.18	**4.38**	**3.41**
**Coc_13**	8	6.25	5.63	5.78	5.83	**7.20**	**−3.43**	**−0.86**
**Average of relative error values**						21.18%	**6.95%**	**5.33%**

## Data Availability

The data used to support the findings of this study can be made available by the corresponding author upon request.
